# Development, Validity, and Reliability of the Primary Eye Care Questionnaire (PECQ) Among Optometrists in Malaysia

**DOI:** 10.7759/cureus.71685

**Published:** 2024-10-17

**Authors:** Prema Muthiah, Sharanjeet Sharanjeet-Kaur, Mohd Izzuddin Hairol, Saadah Mohamed Akhir, Sumithira Narayanasamy

**Affiliations:** 1 Optometry and Vision Science Program, Centre for Community Health Studies (ReaCH), Faculty of Health Sciences, Universiti Kebangsaan Malaysia (UKM), Kuala Lumpur, MYS

**Keywords:** construct validity, face validation, optometrist, optometry services, primary eye care, questionnaire, questionnaire survey, reliability, scope of practice, validity

## Abstract

Introduction: Visual impairment and vision loss are preventable or treatable if there is access to eyecare services for early detection and if proper interventions are given. Optometrists play a vital role in the early detection of eye and visual-related issues. As healthcare professionals, optometrists are trained to provide comprehensive eye and vision care encompassing dispensing and refraction, detection of abnormalities in the vision and eye, and the rehabilitation of conditions of the visual system, as suggested by the World Council of Optometry.In the current practice of primary eye care, optometrists are not fully utilized and are mostly restricted to refractive correction. Therefore, the optometrists’ practice level, scope, and challenges to practice primary eye care must be identified before strengthening optometrists' role in the community.

Objective: The present study aimed to develop and validate a questionnaire to determine the contributing factors and challenges to the practice of primary eye care by optometrists in Malaysia.

Methods: The Primary Eye Care Questionnaire (PECQ) was developed through an extensive literature search to identify the domains and to construct the items. The items were then scrutinized further through focus group discussions with experts (n = 10) from various sectors of eye care. Content validation was conducted with ophthalmologists and optometrists as panels (n = 10), followed by face validation (n = 10) by optometrists from the public and private sectors. Construct validation was performed with exploratory factor analysis and reliability was evaluated by using Cronbach's alpha coefficient and the test-retest reliability.

Results: In total, the PECQ questionnaire was constructed with 123 items under three main domains: contributing factors, challenges, and continuous education. The content validity index (CVI) for each item (I-CVI) score ranged from 0.9 to 1.0 for each item and the CVI for average scale (S-CVI/Ave) was above 0.8 for each domain, with internal consistency of 0.948 and Cronbach's alpha coefficient of 0.971.

Conclusion: The PECQ is a valid and reliable tool for assessing the optometrists’ scope of practice, challenges, and contributing factors to the practice of primary eye care.

## Introduction

The International Agency for the Prevention of Blindness (IAPB) has predicted that with population growth and aging, the number of people with vision loss will increase from 1.1 billion in 2020 to 1.7 billion people by 2050 [1,2]. It was estimated that over 90% of vision loss is avoidable [3], with uncorrected refractive error identified as the leading cause of vision loss. The uncorrected refractive error caused near vision loss in 510 million people and distance vision loss in 161 million people [3]. Whereas, cataract was identified as the second most common cause of vision loss, affecting about 100 million people [1].

A world report in Vision 2020 estimated that 43.3 million people were diagnosed with blindness due to diabetic retinopathy, glaucoma, and age-related macular degeneration (AMD) accounting for 4.4 million, 7.8 million, and 8.1 million people, respectively [2,3]. In Malaysia, the Malaysian National Eye Survey II (NES II) reported the prevalence of blindness, severe visual impairment, and moderate visual impairment was 1.2%, 1.0%, and 5.9%, respectively, out of which 86.3% of the causes of blindness were avoidable [3]. Therefore, undeniably, blindness and visual impairment are major global eye health issues that need immediate attention.

As an effort to reduce preventable visual impairment, a revamp in the healthcare system is therefore necessary at all levels, starting from promoting health and preventing disease through early detection and treatment. Optometrists play a crucial role in the detection of uncorrected refractive error, glaucoma, and diabetic retinopathy through early screening. Optometrists are primary eye care professionals who are the frontliners of vision and eye care. Optometrists have competencies in dispensing, refracting, prescribing for refractive conditions, and detection of diseases or abnormalities of the eye. The role of optometrists in the community is essential as they play a vital role in community healthcare services, mainly in providing clinical services and eye care education [4].

Given that a high percentage of avoidable sight loss was the consequence of inequity and lack of access to eyecare services, measures must be taken to reduce this preventable vision loss. Primary eye care is a crucial component of comprehensive eye care that focuses on reducing ocular morbidity as well as preventing blindness and visual impairment [5]. Also, primary eye care optometrists are three times less likely to make false-positive referrals as compared to general practitioners [6]. Even in asymptomatic patients, optometrists were able to identify the pathology or risk necessitating referral for ophthalmological evaluation [7].

Therefore, in promoting the quality and effectiveness of primary eye care, the optometrist must be equipped with the necessary competency to detect abnormalities to make necessary and timely referrals to relevant professionals for management. The NES II suggested increased patient education and further expansion of ophthalmological services in Malaysia to reduce the rate of avoidable blindness [3]. The role of an optometrist in the primary healthcare system will improve the early diagnosis and timely appropriate referral, potentially reducing the progression of an eye condition [8,9].

Therefore, an eye care model with an emphasized role of optometrists is crucial for this. Prior to the implementation of the optometrist's role in the community, the practice level, scope, and challenges faced by optometrists in the community must be identified. However, there is a lack of questionnaires to identify optometrists' scope of practice, challenges, and contributing factors to the practice of primary eye care. Therefore, the objective of this study was to develop and validate a questionnaire to determine the contributing factors and challenges to the practice of primary eye care by optometrists in Malaysia. Questionnaire-based studies are important tools in perception and behavioral research studies as questionnaires are used to measure a range of information collected based on participants' responses [10]. A well-designed and validated questionnaire is important to establish the quality and scientific worth of any questionnaire-based information. Therefore, questionnaire validation is a crucial step to ensure quality responses and results [11]. Therefore, the Primary Eye Care Questionnaire (PECQ) can serve as the baseline assessment of the optometrist's scope of practice, challenges, and contributing factors to the practice of primary eye care.

## Materials and methods

**Figure 1:**
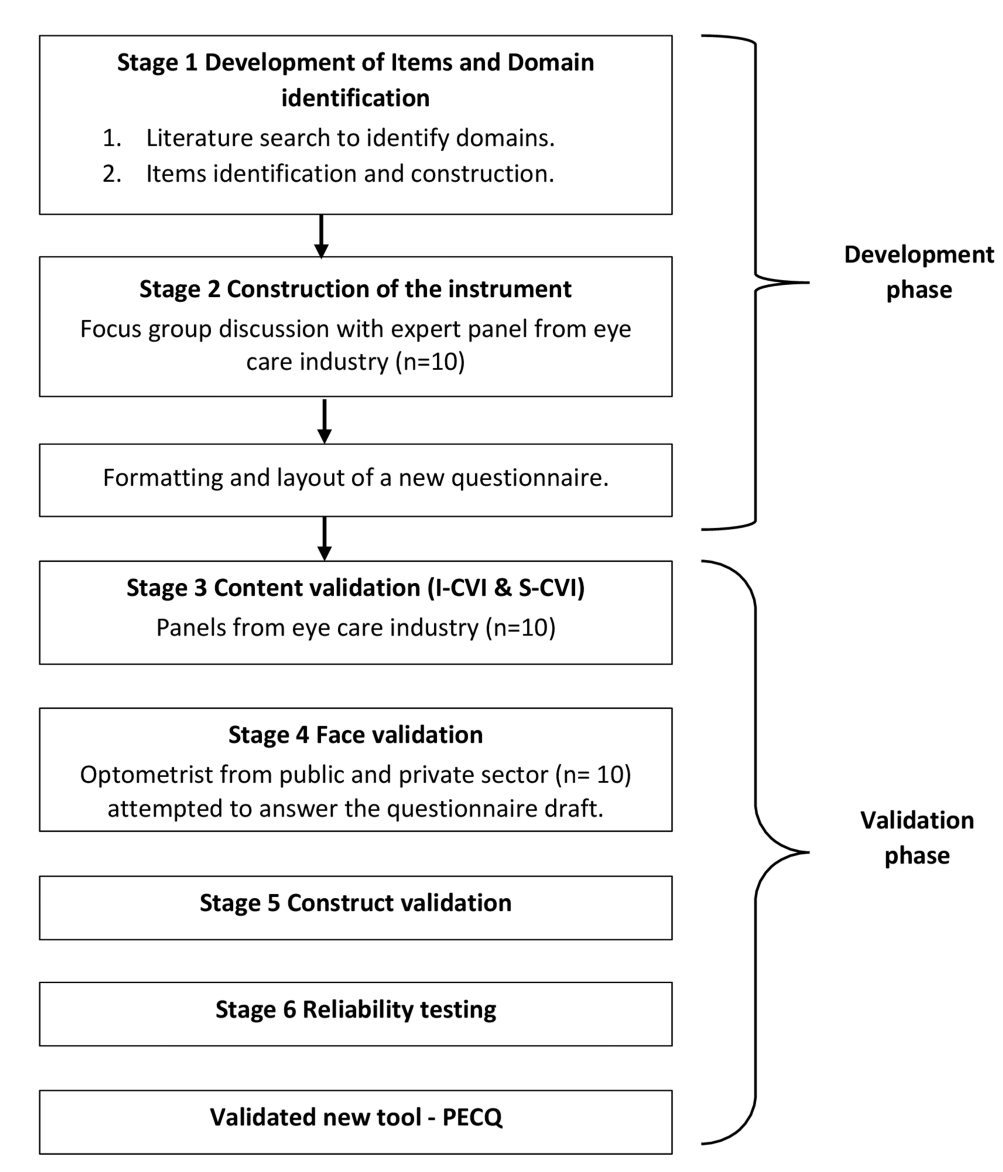
Flowchart of construction, validation, and reliability of the questionnaire. PECQ: Primary Eye Care Questionnaire; I-CVI: item-level content validity index; S-CVI: scale content validity index.

This study elaborates on the development and validation of a new questionnaire on the primary eye care practice scope, its contributing factors, and the challenges faced by optometrists. The main steps and results involved in the construction, validation, and reliability of the questionnaire are summarized in Figure 1.

Questionnaire development

The development of the self-administered questionnaire process took place in six stages: stage 1, item development; stage 2, instrument construction; stage 3, content validation; stage 4, face validation; stage 5, construct validation; and stage 6, reliability testing.

Stage 1: Development of Items and Domain Identification

A new set of questionnaires was developed through a detailed literature search. A literature search was performed to determine the optometrist’s scope of practice, contributing factors, and challenges to the practice of primary eye care. The databases that were selected and searched were PubMed, PubMed Central (PMC), Scopus, EBSCO, Medical Literature Analysis and Retrieval System Online (MEDLINE), OVID, and Web of Science (WoS). The keywords used for the search were “Optometr*,” “Optician*,” “Ophthalmic practitioner*,” “primary eye care,” “scope of practice,” and “challenges.”

Stage 2: Construction of the Instrument

The established domains and items were validated by assessing their relevance, level, and scope of practice consistent with the practice of optometry in Malaysia through a focus group discussion (FGD) conducted online. The objective of the FGD was to identify the suitability of the items to be included in the questionnaire. The FGD included 10 panel experts from the eye care sector in Malaysia, comprising ophthalmologists and optometrists from both the private and public sectors. The number of experts for content validation should be between six to 10 [12]. The inclusion criteria of the panel experts were that the panels must have served in the eye care sector in Malaysia for at least 10 years and have provided consent to participate in the FGD. The domains and items identified and derived through the literature search were screened and checked by the experts through FGD. The panel's comments on the relevance of each identified item were recorded, transcribed, and analyzed by using a thematic analysis.

The first draft of the questionnaire was prepared in Bahasa Malaysia and English language [13,14]. The questionnaire consisted of three main domains: contributing factors, continuing education, and challenges. There were 10 subdomains under contributing factors: the scope of primary eye care, optometry procedures, optometry services, diagnostic instrumentations and drugs, management, referral and training, and community services. The second subdomain of continuous education consisted of the relevance/importance, areas, and fellowship. The third subdomain of challenges consisted of challenges in practicing primary eye care and attending continuous education.

Stage 3: Content Validation

Content validity refers to the extent to which an item on a questionnaire is relevant and representative of the targeted construct [15]. This was measured through the content validity index (CVI), which consists of two forms, the CVI for each item (I-CVI) and the CVI for the average scale (S-CVI/Ave) [15]. The CVI included the appraisal of each item on its relevance and representativeness by the expert’s panel. The first draft of the questionnaire was assessed for CVI by 10 experts who agreed to participate in the validation process [16]. The experts are comprised of ophthalmologists, optometrists, and academics from the private and public sectors with experience in the eye care sector for a minimum of 10 years. These experts were different individuals from those who participated in FGD in stage 2 of questionnaire development. Each expert received the study objective and the consent form through their electronic mail, whereby they reverted the email with the signed copy of consent in agreement to participate in the study. The drafted questionnaire was then emailed to the experts, and they were required to evaluate each item of the questionnaire based on a four-point Likert scale (1 to 4, where 1 = the item is not relevant to the measured domain, and 4 = the item is highly relevant to the measured domain) [17]. The experts were required to respond via email within 14 to 21 days and were given two reminders in between this period. The experts were also encouraged to provide comments, suggestions, or feedback to improve the item’s clarity, relevance, and overall construction. The questionnaire was then edited and emailed back to the experts for confirmation. Once confirmed, the experts’ responses were then analyzed to identify the content validity.

For content validity calculation purposes, the relevance rating was denoted as “1” if the relevance score by experts was 3 or 4 and the relevance rating was “0” if the relevance score was 1 or 2. With that definition, I-CVI was calculated by dividing the total number of experts agreeing with the item, recorded as “1” by the total number of experts, which is 10 [18]. On the other hand, the S-CVI average (S-CVI/Ave) was also calculated. S-CVI/Ave is the mean of the I-CVI scores for all items on the scale or the average of proportion relevance scored by all experts. The mean of relevance rating by individual experts is called the proportion relevant. S-CVI/Ave was determined by dividing the sum of I-CVI scores by the number of items. The accepted I-CVI value for an expert sample of 10 was at least 0.78 [18]. A rating of S-CVI/universal agreement (UA) ≥ 0.8 and an S-CVI/Ave ≥ 0.9 have excellent content validity [19].

The UA score was also calculated to determine the rate of agreement between the experts on content validity. Therefore, UA is rated “1” when the item achieved 100% agreement from all experts and “0” if otherwise. With that, the scale-level content validity index based on the universal agreement method (S-CVI/UA) is calculated by dividing the sum of UA scores by the total number of items in each domain.

The kappa coefficient of agreement (K*) was calculated to eliminate the random chance agreement by the experts in determining the CVI. K* is an important measure as it offers additional information beyond proportion agreement because it removes random chance agreement [20]. Kappa statistic is a consensus index of inter-rater agreement that adjusts for chance agreement [19]. For this, the data variability was first calculated by using the proportion agreement (Pc) for each item utilizing the following formula:

Pc = [N!/A!(N − A)!] * 0.5N.

Where, N = number of experts in a panel, and A = number of experts who agreed that the item is relevant [13].

This was followed by computing the K* by entering the numerical values of the probability of chance agreement (Pc) and CVI of each item (I-CVI) in the following formula:

K = (I-CVI − Pc)/(1 − Pc).

K* value above 0.74 is considered excellent, between 0.60 and 0.74 is good, and the ones between 0.40 and 0.59 are rated as fair [19].

Stage 4: Face Validation

The face validation of a questionnaire is tested with the face validity index (FVI). An FVI test was conducted with selected optometrists to determine the practicality, readability, lexical clarity, and formatting style of the items [21]. This was done to ensure the optometrist understood the item the way it was meant to be comprehended. The respondents were encouraged to actively express their feedback as they attempted to answer the questionnaire draft, to identify misinterpretations and confusing and difficult items.

The minimum acceptable number of respondents was 10 [15]. Therefore, 10 optometrists, five each from the private sector and public sector, were included. The questionnaire and instructions were sent out through email to all the respondents. Each item was reviewed on its clarity and comprehension based on a four-point rating scale: 1 = the item is not clear and not understandable; 4 = the item is very clear and understandable. Their feedback was used to improve the questionnaire further. The items were either edited, removed, or remained unchanged based on the feedback from the optometrists on their understanding of the item structure and language.

Similar to CVI, there were two forms of FVI calculated, the FVI for item (I-FVI) and the FVI for scale (S-FVI). The average of the I-FVI scores for all the items on the scale (S-FVI/Ave) and the proportion of items on the scale that achieved a clarity and comprehension scale of 3 or 4 by all respondents (S-FVI/UA) was then calculated. For the calculation of FVI purposes, the clarity and comprehension rating must be denoted as “1” (a scale of 3 or 4) or “0” (a scale of 1 or 2). The accepted FVI was 0.83 or more (24). This process produced the third draft of the questionnaire.

Stage 5: Construct Validity

The minimum sample size required to make accurate inferences in a small population using the exploratory factor analysis (EFA) is 100 [16]. This sample size should be added with a 20% drop-out rate. Therefore, the sample size required for construct validity was 120 optometrists who fulfilled the inclusion criteria. The inclusion criteria included the following: the optometrist must be a Malaysian, practicing in optometry services, and registered as an optometrist with the Malaysian Optics Council (MOC).

The determination of the underlying structure of the questionnaire items was tested by using the EFA. The determination of the adequacy of the EFA was calculated through the analysis of Bartlett’s test and the Kaiser-Meyer-Olkin (KMO) measure. The KMO statistics are between 0 and 1 and the values closer to 1 indicate greater adequacy of the factor analysis (KMO ≥ 0.6 = low adequacy, KMO ≥ 0.7 = medium adequacy, KMO ≥ 0.8 = high adequacy, KMO ≥ 0.9 = very high adequacy) [22]. A principal component factor analysis utilizing varimax rotation by fixing the number of factors to extract and a cut-off point of 0.3 was used for commonalities with ideal commonalities being 0.7 or above [23]. Factor loadings close to -1 or 1 indicate that the factor strongly influences the variable, whereas loadings close to 0 indicate that the factor has a weak influence on the variable, and thus should be removed. The total variance is the sum of variances of all individual principal components. The fraction of variance explained by a principal component is the ratio between the variance of that principal component and the total variance. Variance explained by factor analysis should not be less than 60% [24].

Stage 6: Reliability Test

The questionnaire's reliability was evaluated by using Cronbach’s alpha coefficient and the test-retest reliability [24]. Internal consistency (IC) of the domains (i.e., contributing factors, challenges, and continuous education) was assessed by using Cronbach’s alpha coefficient for the Likert scale. The homogeneity of the question items in each domain was evaluated utilizing Cronbach’s alpha coefficient. Cronbach’s alpha coefficient values of equal or more than 0.7 were considered acceptable to excellent [10,25], indicating the internal consistency of the PECQ.

The test-retest measured the stability and consistency of the respondents when the PECQ was administered again after two weeks [21]. The same group of optometrists were administered with PECQ after two weeks following the response from the first administration. This time frame was chosen to reduce the impact of potential confounding factors that could affect the findings, such as the memory effect. The test-retest reliability was assessed utilizing the intraclass correlation coefficient (ICC), Bland-Altman plot, and standard error of measurement. The confidence interval (CI) of 95% was used. The sample size was calculated using a web-based sample size calculator for reliability studies [23]. The calculator is accessible at https://wnarifin.github.io/ssc/ssicc.html, whereby the minimum acceptable reliability ICC was set at 0.7, the expected ICC was 0.85, the significance level was 0.05, the power was 80%, and the minimum number of respondents was set at 10. The expected drop-out rate was set at 10% [23]. The calculation using the web-based calculator showed the expected sample size of 30 optometrists.

Ethical approval

This study was approved by the Research Ethics Committee, Universiti Kebangsaan Malaysia (JEP-2020-575) and the Medical Research and Ethics Committee (MREC), Ministry of Health Malaysia (MOH) (NMRR-21-1734-60845).

## Results

Stage 1: Development of items and domain identification

The literature search generated seven domains, including scope of practice, instruments, practice settings, specialty field, challenges in practicing optometry, fellowships, and continuous professional development. These domains and search items were compiled in an Excel sheet (Microsoft Corporation, Redmond, WA) for the FGD panels.

Stage 2: Construction of the instrument

The included domains were then constructed as items for the questionnaire draft were formed. A total of seven domains were then merged to form six domains and 152 items in the first draft of the questionnaire. Using this as a guide, the FGD panel’s opinions were recorded through an online discussion. The descriptive analysis of the FGD panel's demographics is summarized in Table 1.

**Table 1:** Demographic of respondents for focus group panels.

	Characteristics	n	Mean ± SD/Percentage
Mean age	Age (years)	10	41.5 ± 6.04 years
Gender	Male	2	20%
	Female	8	80%
Race	Malay	5	50%
	Chinese	4	40%
	Indian	1	10%
Sector	Private	8	80%
	Public	2	20%
Experience in the eye care industry	10-15 years	3	30%
	15-20 years	5	50%
	>20 years	2	20%
Profession	Ophthalmologist	1	10%
	Optometrist	9	90%

**Figure 2:**
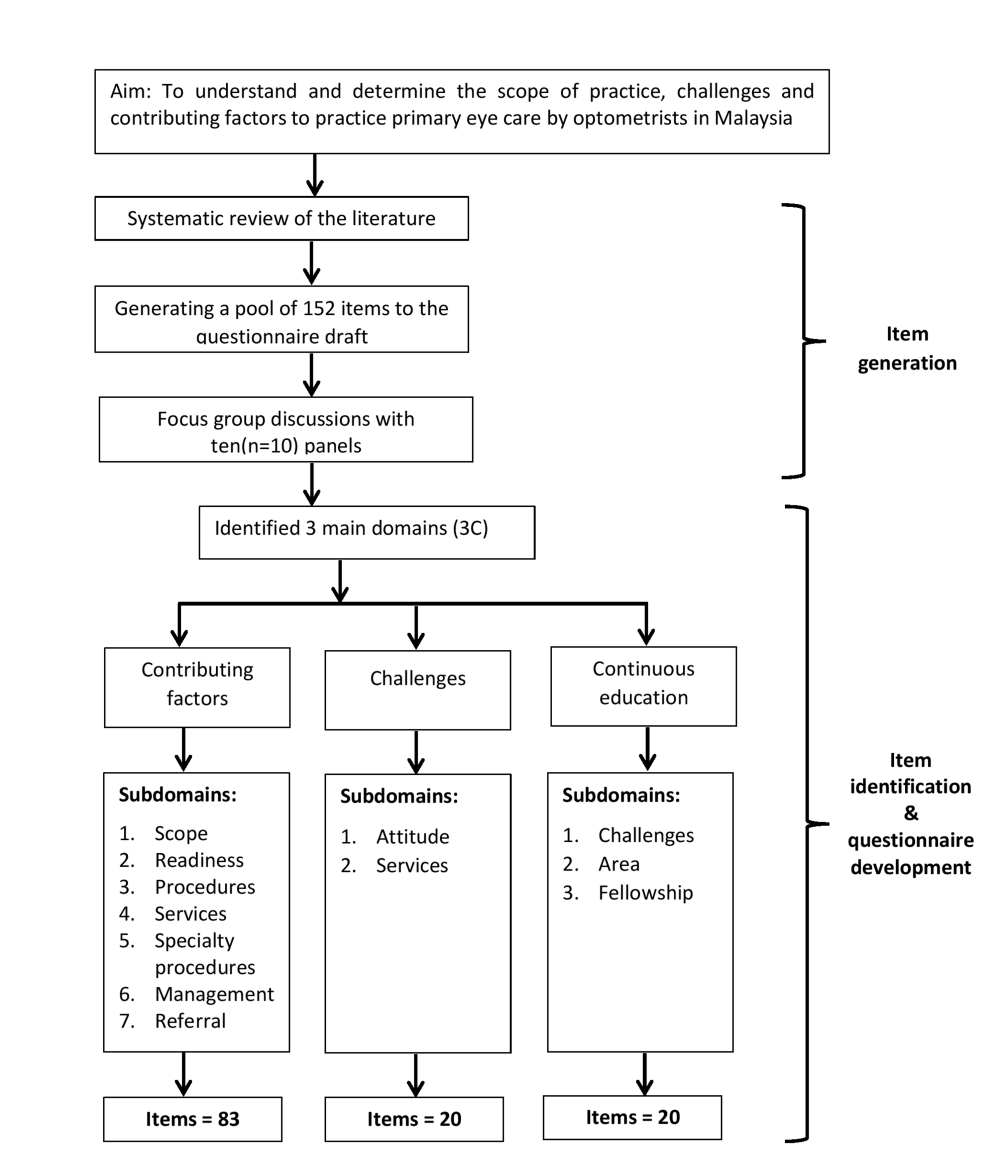
Overview of the literature selection process.

The FGD panels reviewed the items for their relevance and scope for the practice of optometry in Malaysia. The relevant items were then selected and the non-relevant were deleted. The first draft of the questionnaire was developed in English and Bahasa Malaysia and consisted of 123 items in total. Three main domains were identified: contributing factors, challenges, and continuous education. The contributing factors, whereby the domain had seven subdomains with 83 items, the challenges domain had two subdomains with 20 items, and the continuous education domain consisted of three subdomains with 20 items. Figure 2 summarizes the finalized questionnaire items that were decided in FGD.

Stage 3: Content validation

The first draft of the questionnaire was evaluated for content validity by a group of 10 experts. The experts consist of ophthalmologists and optometrists to evaluate the items for content, language, and relevance. The descriptive analysis of the respondents is summarized in Table 2.

**Table 2:** Demographic of content validation panels.

	Characteristics	n	Mean ± SD/Percentage
Mean age	Age (years)	10	41.1 ± 6.56 years
Gender	Male	2	20%
	Female	8	80%
Race	Malay	4	40%
	Chinese	5	50%
	Indian	1	10%
Sector	Private	7	70%
	Public	3	30%
Experience in the eye care industry	10-15 years	5	50%
	15-20 years	1	10%
	>20 years	4	40%
Profession	Ophthalmologist	2	20%
	Optometrist	8	80%

The CVI for each item (I-CVI) and the CVI for average scale (S-CVI/Ave), the scale-level CVI based on universal agreement (S-CVI/UA), probability of change agreement (Pc), and modified kappa (K) were calculated. Table 3 summarizes the expert agreement, I-CVI value, interpretation, UA value by the panel reviewers, probability of change agreement (Pc), and modified kappa (K) for the first domain, which are the contributing factors. The contributing factors domain has seven subdomains and a total of 83 items. From Table 3, it was evident that all items achieved 90-100% expert agreement and I-CVI of 0.9 and above. This indicates the items were clear and relevant. The S-CVI/Ave value for the contributing factors domain was 0.98, indicating excellent content validity. The S-CVI/UA, the scale-level content validity index based on the universal agreement method, was 0.88. These values indicate a good agreement between the items within the domain.

**Table 3:** The content validity for contributing factors domain (domain 1) with interpretation. I-CVI: item-level content validity index; UA: universal agreement; Pc: probability of change agreement.

Subdomain	Items	Experts in agreement	I-CVI	I-CVI interpretation	UA	Pc	Modified kappa	Interpretation
Scope	Item 1	9	0.9	Appropriate	0	0.11	0.89	Good
	Item 2	9	0.9	Appropriate	0	0.11	0.89	Good
	Item 3	10	1	Appropriate	1	0.00	1.00	Excellent
	Item 4	10	1	Appropriate	1	0.00	1.00	Excellent
	Item 5	10	1	Appropriate	1	0.00	1.00	Excellent
	Item 6	10	1	Appropriate	1	0.00	1.00	Excellent
	Item 7	10	1	Appropriate	1	0.00	1.00	Excellent
	Item 8	10	1	Appropriate	1	0.00	1.00	Excellent
Readiness	Item 9	10	1	Appropriate	1	0.00	1.00	Excellent
	Item 10	9	0.9	Appropriate	0	0.11	0.89	Good
	Item 11	10	1	Appropriate	1	0.00	1.00	Excellent
	Item 12	10	1	Appropriate	1	0.00	1.00	Excellent
	Item 13	10	1	Appropriate	1	0.00	1.00	Excellent
Basic procedures	Item 14	10	1	Appropriate	1	0.00	1.00	Excellent
	Item 15	10	1	Appropriate	1	0.00	1.00	Excellent
	Item 16	10	1	Appropriate	1	0.00	1.00	Excellent
	Item 17	10	1	Appropriate	1	0.00	1.00	Excellent
	Item 18	10	1	Appropriate	1	0.00	1.00	Excellent
	Item 19	10	1	Appropriate	1	0.00	1.00	Excellent
	Item 20	10	1	Appropriate	1	0.00	1.00	Excellent
	Item 21	10	1	Appropriate	1	0.00	1.00	Excellent
	Item 22	10	1	Appropriate	1	0.00	1.00	Excellent
	Item 23	10	1	Appropriate	1	0.00	1.00	Excellent
	Item 24	10	1	Appropriate	1	0.00	1.00	Excellent
	Item 25	10	1	Appropriate	1	0.00	1.00	Excellent
	Item 26	10	1	Appropriate	1	0.00	1.00	Excellent
	Item 27	10	1	Appropriate	1	0.00	1.00	Excellent
	Item 28	10	1	Appropriate	1	0.00	1.00	Excellent
	Item 29	10	1	Appropriate	1	0.00	1.00	Excellent
	Item 30	10	1	Appropriate	1	0.00	1.00	Excellent
	Item 31	10	1	Appropriate	1	0.00	1.00	Excellent
Services	Item 32	10	1	Appropriate	1	0.00	1.00	Excellent
	Item 33	10	1	Appropriate	1	0.00	1.00	Excellent
	Item 34	10	1	Appropriate	1	0.00	1.00	Excellent
	Item 35	10	1	Appropriate	1	0.00	1.00	Excellent
	Item 36	10	1	Appropriate	1	0.00	1.00	Excellent
	Item 37	10	1	Appropriate	1	0.00	1.00	Excellent
	Item 38	10	1	Appropriate	1	0.00	1.00	Excellent
	Item 39	10	1	Appropriate	1	0.00	1.00	Excellent
	Item 40	9	0.9	Appropriate	0	0.11	0.89	Good
	Item 41	9	0.9	Appropriate	0	0.11	0.89	Good
Specialty procedures	Item 42	10	1	Appropriate	1	0.00	1.00	Excellent
	Item 43	10	1	Appropriate	1	0.00	1.00	Excellent
	Item 44	10	1	Appropriate	1	0.00	1.00	Excellent
	Item 45	10	1	Appropriate	1	0.00	1.00	Excellent
	Item 46	10	1	Appropriate	1	0.00	1.00	Excellent
	Item 47	10	1	Appropriate	1	0.00	1.00	Excellent
	Item 48	10	1	Appropriate	1	0.00	1.00	Excellent
	Item 49	10	1	Appropriate	1	0.00	1.00	Excellent
	Item 50	10	1	Appropriate	1	0.00	1.00	Excellent
	Item 51	10	1	Appropriate	1	0.00	1.00	Excellent
	Item 52	10	1	Appropriate	1	0.00	1.00	Excellent
	Item 53	10	1	Appropriate	1	0.00	1.00	Excellent
	Item 54	9	0.9	Appropriate	0	0.11	0.89	Good
	Item 55	9	0.9	Appropriate	0	0.11	0.89	Good
	Item 56	10	1	Appropriate	1	0.00	1.00	Excellent
	Item 57	10	1	Appropriate	1	0.00	1.00	Excellent
	Item 58	10	1	Appropriate	1	0.00	1.00	Excellent
Management	Item 59	9	0.9	Appropriate	0	0.11	0.89	Good
	Item 60	10	1	Appropriate	1	0.00	1.00	Excellent
	Item 61	10	1	Appropriate	1	0.00	1.00	Excellent
	Item 62	10	1	Appropriate	1	0.00	1.00	Excellent
	Item 63	10	1	Appropriate	1	0.00	1.00	Excellent
	Item 64	10	1	Appropriate	1	0.00	1.00	Excellent
	Item 65	10	1	Appropriate	1	0.00	1.00	Excellent
	Item 66	10	1	Appropriate	1	0.00	1.00	Excellent
	Item 67	10	1	Appropriate	1	0.00	1.00	Excellent
	Item 68	10	1	Appropriate	1	0.00	1.00	Excellent
Referral	Item 69	10	1	Appropriate	1	0.00	1.00	Excellent
	Item 70	10	1	Appropriate	1	0.00	1.00	Excellent
	Item 71	10	1	Appropriate	1	0.00	1.00	Excellent
	Item 72	10	1	Appropriate	1	0.00	1.00	Excellent
	Item 73	9	0.9	Appropriate	0	0.11	0.89	Good
	Item 74	10	1	Appropriate	1	0.00	1.00	Excellent
	Item 75	10	1	Appropriate	1	0.00	1.00	Good
	Item 76	10	1	Appropriate	1	0.00	1.00	Excellent
	Item 77	10	1	Appropriate	1	0.00	1.00	Good
	Item 78	10	1	Appropriate	1	0.00	1.00	Excellent
	Item 79	10	1	Appropriate	1	0.00	1.00	Good
	Item 80	10	1	Appropriate	1	0.00	1.00	Excellent
	Item 81	10	1	Appropriate	1	0.00	1.00	Good
	Item 82	10	1	Appropriate	1	0.00	1.00	Excellent
	Item 83	9	0.9	Appropriate	0	0.11	0.89	Good

Domain 2 is on the challenges and it has two subdomains with 20 items (item 84 to item 103). Table 4 summarizes the expert agreement, I-CVI value, interpretation, UA value by the panel reviewers, probability of change agreement (Pc), and modified kappa (K) for domain 2. From Table 4, it was evident that all items achieved 100% expert agreement, except item 95 achieved 90%, which is still good. The I-CVI of each item was 0.9 and above, indicating each item was relevant and clear. The S-CVI/Ave value for the second domain was 0.99, indicating excellent content validity. The S-CVI/UA, the scale-level content validity index based on the universal agreement method, was 0.95. These values indicate excellent agreement between the items within the domain.

**Table 4:** The content validity for challenges domain (domain 2) with interpretation. I-CVI: item-level content validity index; UA: universal agreement; Pc: probability of change agreement.

Subdomain	Items	Experts in agreement	I-CVI	I-CVI interpretation	UA	Pc	Modified kappa	Interpretation
Attitude	Item 84	10	1	Appropriate	1	0.00	1.00	Excellent
	Item 85	10	1	Appropriate	1	0.00	1.00	Excellent
	Item 86	10	1	Appropriate	1	0.00	1.00	Excellent
	Item 87	10	1	Appropriate	1	0.00	1.00	Excellent
	Item 88	10	1	Appropriate	1	0.00	1.00	Excellent
	Item 89	10	1	Appropriate	1	0.00	1.00	Excellent
	Item 90	10	1	Appropriate	1	0.00	1.00	Excellent
	Item 91	10	1	Appropriate	1	0.00	1.00	Excellent
	Item 92	10	1	Appropriate	1	0.00	1.00	Excellent
	Item 93	10	1	Appropriate	1	0.00	1.00	Excellent
	Item 94	10	1	Appropriate	1	0.00	1.00	Excellent
	Item 95	9	0.9	Appropriate	0	0.11	0.89	Good
Services	Item 96	10	1	Appropriate	1	0.00	1.00	Excellent
	Item 97	10	1	Appropriate	1	0.00	1.00	Excellent
	Item 98	10	1	Appropriate	1	0.00	1.00	Excellent
	Item 99	10	1	Appropriate	1	0.00	1.00	Excellent
	Item 100	10	1	Appropriate	1	0.00	1.00	Excellent
	Item 101	10	1	Appropriate	1	0.00	1.00	Excellent
	Item 102	10	1	Appropriate	1	0.00	1.00	Excellent
	Item 103	10	1	Appropriate	1	0.00	1.00	Excellent

The third domain is continuous education with three subdomains and 20 items (item 104 to item 123). Table 5 summarizes the expert agreement, I-CVI value, interpretation, UA value by the panel reviewers, probability of change agreement (Pc), and modified kappa (K) for domain 3. As shown in Table 5, all items in the third domain achieved more than 90% of expert agreement. The I-CVI of each item was 0.9 and above, indicating each item was relevant and clear. The S-CVI/Ave value for the third domain was 0.98, indicating excellent content validity. The S-CVI/UA, the scale-level content validity index based on the universal agreement method, was 0.85. These values indicate a good agreement between the items within the domain.

**Table 5:** The content validity for continuous education domain (domain 3) with interpretation. I-CVI: item-level content validity index; UA: universal agreement; Pc: probability of change agreement.

Subdomain	Items	Experts in agreement	I-CVI	I-CVI interpretation	UA	Pc	Modified kappa	Interpretation
Challenges	Item 104	10	1	Appropriate	1	0.00	1.00	Excellent
	Item 105	10	1	Appropriate	1	0.00	1.00	Excellent
	Item 106	10	1	Appropriate	1	0.00	1.00	Excellent
	Item 107	10	1	Appropriate	1	0.00	1.00	Excellent
	Item 108	9	0.9	Appropriate	0	0.11	0.89	Good
	Item 109	10	1	Appropriate	1	0.00	1.00	Excellent
Area	Item 110	10	1	Appropriate	1	0.00	1.00	Excellent
	Item 111	10	1	Appropriate	1	0.00	1.00	Excellent
	Item 112	10	1	Appropriate	1	0.00	1.00	Excellent
	Item 113	10	1	Appropriate	1	0.00	1.00	Excellent
	Item 114	10	1	Appropriate	1	0.00	1.00	Excellent
	Item 115	9	0.9	Appropriate	0	0.11	0.89	Good
	Item 116	10	1	Appropriate	1	0.00	1.00	Excellent
Fellowship	Item 117	10	1	Appropriate	1	0.00	1.00	Excellent
	Item 118	10	1	Appropriate	1	0.00	1.00	Excellent
	Item 119	10	1	Appropriate	1	0.00	1.00	Excellent
	Item 120	10	1	Appropriate	1	0.00	1.00	Excellent
	Item 121	10	1	Appropriate	1	0.00	1.00	Excellent
	Item 122	10	1	Appropriate	1	0.00	1.00	Excellent
	Item 123	9	0.9	Appropriate	0	0.11	0.89	Good

Stage 4: Face validation

The face validity was performed with 10 optometrists. The optometrists were selected from both the public and private sectors. The descriptive analysis of the respondents of face validation is summarized in Table 6.

**Table 6:** Demographic of respondents for face validity.

	Characteristics	n	Mean ± SD/Percentage
Mean age	Age (years)	10	41.1 ± 6.6 years
Gender	Male	2	20%
	Female	8	80%
Race	Malay	4	40%
	Chinese	5	50%
	Others (Bumiputera Sabah)	1	10%
Sector	Private	5	50%
	Public	5	50%
Education level in optometry	Degree	1	10%
	Masters	3	30%
	PhD	6	60%
Experience in the eye care industry	10-15 years	7	70%
	15-20 years	1	10%
	>20 years	2	20%

In face validation, the respondents were asked to comment on the clarity, simplicity of the question structure, and appropriateness of the language and font, and neatness of the questionnaire. There were two corrections made in terms of grammatical errors for item 27 and item 58. There were no other corrections made to the questionnaire in both languages. The FVI for each item (I-FVI) ranged between 0.9 and 1.00, which was more than the expected value (>0.83). The average of the I-FVI scores for all the items on the scale (S-FVI/Ave) and the proportion of items on the scale that achieved clarity and comprehension was also more than 0.83 for all three domains. Table 7 illustrates the face validation of each item’s clarity and comprehension for domain 1, Table 8 for domain 2, and Table 9 for domain 3.

**Table 7:** The face validation of each item’s clarity and comprehension for items in domain 1. I-CVI: item-level content validity index; UA: universal agreement; I-FVI: face validity index for each item; S-CVI/Ave: content validity index for average scale; S-CVI/UA: scale-level content validity index based on universal agreement.

Subdomain	Items	Experts in agreement	I-FVI	I-CVI interpretation	UA	S-CVI/Ave	S-CVI/UA
Scope	Item 1	9	0.9	Good	0	0.96	0.83
	Item 2	9	0.9	Excellent	0		
	Item 3	10	1	Excellent	1		
	Item 4	10	1	Excellent	1		
	Item 5	10	1	Excellent	1		
	Item 6	10	1	Excellent	1		
	Item 7	10	1	Excellent	1		
	Item 8	10	1	Excellent	1		
Readiness	Item 9	9	0.9	Good	0		
	Item 10	10	1	Excellent	1		
	Item 11	10	1	Excellent	1		
	Item 12	10	1	Excellent	1		
	Item 13	10	1	Excellent	1		
Basic procedures	Item 14	9	0.9	Good	1		
	Item 15	10	1	Excellent	1		
	Item 16	10	1	Good	1		
	Item 17	10	1	Excellent	1		
	Item 18	9	0.9	Good	0		
	Item 19	9	0.9	Good	0		
	Item 20	10	1	Excellent	1		
	Item 21	9	0.9	Good	0		
	Item 22	10	1	Excellent	1		
	Item 23	10	1	Excellent	1		
	Item 24	10	1	Excellent	1		
	Item 25	10	1	Excellent	1		
	Item 26	10	1	Excellent	1		
	Item 27	10	1	Excellent	1		
	Item 28	10	1	Excellent	1		
	Item 29	10	1	Excellent	1		
	Item 30	10	1	Excellent	1		
	Item 31	10	1	Excellent	1		
Services	Item 32	10	1	Excellent	1		
	Item 33	10	1	Excellent	1		
	Item 34	10	1	Excellent	1		
	Item 35	10	1	Excellent	1		
	Item 36	9	0.9	Good	0		
	Item 37	9	0.9	Good	0		
	Item 38	10	1	Excellent	1		
	Item 39	10	1	Excellent	1		
	Item 40	10	1	Excellent	1		
	Item 41	10	1	Excellent	1		
Specialty procedures	Item 42	10	1	Excellent	1		
	Item 43	10	1	Excellent	1		
	Item 44	10	1	Excellent	1		
	Item 45	10	1	Excellent	1		
	Item 46	10	1	Excellent	1		
	Item 47	10	1	Excellent	1		
	Item 48	10	1	Excellent	1		
	Item 49	10	1	Excellent	1		
	Item 50	10	1	Excellent	1		
	Item 51	10	1	Excellent	1		
	Item 52	10	1	Excellent	1		
	Item 53	10	1	Excellent	1		
	Item 54	10	1	Excellent	1		
	Item 55	10	1	Excellent	1		
	Item 56	10	1	Excellent	1		
	Item 57	10	1	Excellent	1		
	Item 58	10	1	Excellent	1		
Management	Item 59	9	0.9	Good	0		
	Item 60	10	1	Excellent	1		
	Item 61	10	1	Excellent	1		
	Item 62	10	1	Excellent	1		
	Item 63	10	1	Excellent	1		
	Item 64	9	0.9	Good	0		
	Item 65	10	1	Excellent	1		
	Item 66	10	1	Excellent	1		
	Item 67	10	1	Excellent	1		
	Item 68	10	1	Excellent	1		
Referral	Item 69	10	1	Excellent	1		
	Item 70	10	1	Excellent	1		
	Item 71	10	1	Excellent	1		
	Item 72	10	1	Excellent	1		
	Item 73	8	0.8	Good	0		
	Item 74	10	1	Excellent	1		
	Item 75	10	1	Excellent	1		
	Item 76	10	1	Excellent	1		
	Item 77	9	0.9	Good	0		
	Item 78	10	1	Excellent	1		
	Item 79	10	1	Excellent	1		
	Item 80	10	1	Excellent	1		
	Item 81	10	1	Excellent	1		
	Item 82	10	1	Excellent	1		
	Item 83	9	0.9	Good	0		

**Table 8:** The face validation of each item’s clarity and comprehension for items in domain 2. I-CVI: item-level content validity index; UA: universal agreement; I-FVI: face validity index for each item; S-CVI/Ave: content validity index for average scale; S-CVI/UA: scale-level content validity index based on universal agreement.

Subdomain	Items	Experts in agreement	I-FVI	I-CVI interpretation	UA	S-CVI/Ave	S-CVI/UA
Attitude	Item 84	9	0.9	Good	0	0.975	0.85
	Item 85	10	1	Excellent	1		
	Item 86	10	1	Excellent	1		
	Item 87	10	1	Excellent	1		
	Item 88	10	1	Excellent	1		
	Item 89	10	1	Excellent	1		
	Item 90	9	0.9	Good	0		
	Item 91	10	1	Excellent	11		
	Item 92	10	1	Excellent	1		
	Item 93	10	1	Excellent	1		
	Item 94	10	1	Excellent	1		
	Item 95	10	1	Excellent	1		
Services	Item 96	8	0.8	Good	0		
	Item 97	10	1	Excellent	1		
	Item 98	10	1	Excellent	1		
	Item 99	10	1	Excellent	1		
	Item 100	10	1	Excellent	1		
	Item 101	9	0.9	Good	1		
	Item 102	9	0.9	Good	1		
	Item 103	10	1	Excellent	1		

**Table 9:** The face validation of each item’s clarity and comprehension for items in domain 3. I-CVI: item-level content validity index; UA: universal agreement; I-FVI: face validity index for each item; S-CVI/Ave: content validity index for average scale; S-CVI/UA: scale-level content validity index based on universal agreement.

Subdomain	Items	Experts in agreement	I-FVI	I-CVI interpretation	UA	S-CVI/Ave	S-CVI/UA
Challenges	Item 104	10	1	Excellent	1	1	1
	Item 105	10	1	Excellent	1		
	Item 106	10	1	Excellent	1		
	Item 107	10	1	Excellent	1		
	Item 108	10	1	Excellent	1		
	Item 109	10	1	Excellent	1		
Area	Item 110	10	1	Excellent	1		
	Item 111	10	1	Excellent	1		
	Item 112	10	1	Excellent	1		
	Item 113	10	1	Excellent	1		
	Item 114	10	1	Excellent	1		
	Item 115	10	1	Excellent	1		
	Item 116	10	1	Excellent	1		
Fellowship	Item 117	10	1	Excellent	1		
	Item 118	10	1	Excellent	1		
	Item 119	10	1	Excellent	1		
	Item 120	10	1	Excellent	1		
	Item 121	10	1	Excellent	1		
	Item 122	10	1	Excellent	1		
	Item 123	10	1	Excellent	1		

Stage 5: Construct validity

Exploratory factor analysis (EFA) was used to confirm the factorial structure of the questionnaire. A total of 123 items were subjected to EFA to examine the factorial validity of the scale. The Kaiser-Meyer-Olkin (KMO) measure of sampling adequacy was 0.716 and Bartlett’s test of sphericity reached statistical significance (p-value < 0.001) (chi-squared, df = 9849.933,3081). This indicated that the data were suitable for factor analysis. The KMO values for each domain are illustrated in Table 10. All three domains had highly significant sampling, which is adequate for this study.

**Table 10:** The KMO value as a prerequisite for factor analysis.

Domain	Bartlett’s test and the Kaiser-Meyer-Olkin (KMO) measure	Interpretation
Contributing factors	0.704 (p < 0.001)	Medium adequacy and significant sampling
Challenges	0.697 (p < 0.001)	Medium adequacy and significant sampling
Continuous education	0.758 (p < 0.001)	High adequacy and significant sampling

Therefore, the sampling was adequate for factor analysis and the results showed that the factor loading of each item was close to +1 to -1. This indicated that the factor had strongly influenced the variable and loadings were close to “0” indicating that the factor had a weak influence on the variable. The total variance is the sum of variances of all individual principal components. The fraction of variance explained by a principal component is the ratio between the variance of that principal component and the total variance. The variance explained by factor analysis should not be less than 60%. The results from this study showed the total variance explained for each domain was 78.69%, 68.77%, and 70.56%, respectively. This showed that the questionnaire items were adequate and well-represented in each domain. The factor loadings and total variance explained are illustrated in Table 11.

**Table 11:** Factor loading and total variance explained.

Domain	Subdomain	Items	Factor loading	Total variance explained
Contributing factors	Scope	Item 1	0.674	78.69%
		Item 2	0.856	
		Item 3	0.863	
		Item 4	0.89	
		Item 5	0.826	
		Item 6	0.891	
		Item 7	0.71	
		Item 8	0.796	
	Readiness	Item 9	0.816	
		Item 10	0.822	
		Item 11	0.783	
		Item 12	0.799	
		Item 13	0.866	
	Basic procedures	Item 14	0.674	
		Item 15	0.798	
		Item 16	0.817	
		Item 17	0.709	
		Item 18	0.785	
		Item 19	0.784	
		Item 20	0.683	
		Item 21	0.634	
		Item 22	0.801	
		Item 23	0.85	
		Item 24	0.758	
		Item 25	0.812	
		Item 26	0.817	
		Item 27	0.716	
		Item 28	0.767	
		Item 29	0.663	
		Item 30	0.773	
		Item 31	0.787	
	Services	Item 32	0.818	
		Item 33	0.746	
		Item 34	0.702	
		Item 35	0.747	
		Item 36	0.823	
		Item 37	0.736	
		Item 38	0.859	
		Item 39	0.878	
		Item 40	0.853	
		Item 41	0.677	
	Specialty procedures	Item 42	0.756	
		Item 43	0.63	
		Item 44	0.796	
		Item 45	0.778	
		Item 46	0.85	
		Item 47	0.803	
		Item 48	0.624	
		Item 49	0.771	
		Item 50	0.775	
		Item 51	0.811	
		Item 52	0.783	
		Item 53	0.651	
		Item 54	0.837	
		Item 55	0.832	
		Item 56	0.804	
		Item 57	0.861	
		Item 58	0.813	
	Management	Item 59	0.865	
		Item 60	0.803	
		Item 61	0.806	
		Item 62	0.718	
		Item 63	0.705	
		Item 64	0.721	
		Item 65	0.724	
		Item 66	0.739	
		Item 67	0.694	
		Item 68	0.811	
	Referral	Item 69	0.756	
		Item 70	0.795	
		Item 71	0.829	
		Item 72	0.866	
		Item 73	0.894	
		Item 74	0.889	
		Item 75	0.678	
		Item 76	0.762	
		Item 77	0.858	
		Item 78	0.874	
		Item 79	0.89	
		Item 80	0.898	
		Item 81	0.841	
		Item 82	0.86	
		Item 83	0.797	
Challenges	Attitude	Item 84	0.828	68.77%
		Item 85	0.771	
		Item 86	0.874	
		Item 87	0.870	
		Item 88	0.500	
		Item 89	0.688	
		Item 90	0.546	
		Item 91	0.565	
		Item 92	0.525	
		Item 93	0.772	
		Item 94	0.612	
		Item 95	0.677	
	Services	Item 96	0.680	
		Item 97	0.776	
		Item 98	0.654	
		Item 99	0.793	
		Item 100	0.631	
		Item 101	0.486	
		Item 102	0.785	
		Item 103	0.718	
Continuous education	Challenges	Item 104	0.729	70.563%
		Item 105	0.433	
		Item 106	0.593	
		Item 107	0.657	
		Item 108	0.568	
		Item 109	0.636	
	Area	Item 110	0.635	
		Item 111	0.711	
		Item 112	0.872	
		Item 113	0.833	
		Item 114	0.852	
		Item 115	0.788	
		Item 116	0.776	
	Fellowship	Item 117	0.699	
		Item 118	0.686	
		Item 119	0.776	
		Item 120	0.773	
		Item 121	0.838	
		Item 122	0.585	
		Item 123	0.671	

Stage 6: Reliability analysis

The reliability of the questionnaire was evaluated by using Cronbach’s alpha coefficient and the test-retest reliability utilizing a sub-sample of 10% of the sample size (n = 30) of the optometrists, who were asked to respond to the questionnaire. The Cronbach’s alpha value of 0.971 was obtained, indicating an excellent agreement between the respondents. For test-retest reliability, all the optometrists completed the questionnaire for the second time within a period of 14-day intervals, with few reminders in between. Therefore, the response rate achieved was 100%. The intraclass correlation coefficient was 0.948 (p < 0.01), indicating an excellent agreement.

The final version of the validated questionnaire

The final questionnaire (PECQ) consisted of three main domains: contributing factors, continuous education, and challenges. There were seven subdomains for contributing factors, three subdomains for continuous education, and two subdomains for challenges. There were a total of 123 items with a score of 5 on the Likert scale. The first section of the PECQ consisted of demographic characteristics that consisted of 13 questions. The PECQ had an excellent overall reliability and acceptability. The validated questionnaire is now ready to be used for further data collection.

## Discussion

This study aimed to develop and validate a new standardized and reliable questionnaire to assess optometrists' scope of practice, challenges, and contributing factors to the practice of primary eye care in Malaysia. To date, there was no questionnaire to study the optometrist practice and this questionnaire can fill that gap. The PECQ was built in English and Bahasa Malaysia to ensure it would capture the full participation of optometrists since these are two widely spoken and studied languages in Malaysia. The questionnaire was validated amongst the optometrist population, which indicated the questionnaire is relevant to the population. The PECQ provided a unique self-report tool to assess the primary eye care level of practice amongst optometrists and identify the possible factors that hinder the practice of primary eye care.

The PECQ consisted of 123 items, which were divided into three main domains, i.e., contributing factors, challenges, and continuous education. The draft of the items was constructed based on the critical and systematic review of existing literature and assessment tools together with an FGD with the experts in the eye care industry in Malaysia. In the validation phase of PECQ, the developed items were validated by panels selected for their expertise in the various areas of the eyecare industry, which could strengthen the face and content validity of the questionnaire.

The content validity of the PECQ was calculated by using the I-CVI and S-CVI/Ave. Both values showed that the items were valid. The total number of experts considered adequate for content validation ranges from a minimum of three to a maximum of 10 [7,16]. This study included a panel of 10 experts, which is excellent for determining the agreement between the experts. An I-CVI of 0.78 or higher is considered excellent. The I-CVIs of all items in the PECQ ranged from 0.9 to 1.00. The S-CVI, which assessed the relevancy of the overall questionnaire, was 0.98, indicating the high content validity of the questionnaire. The modified kappa values for all items were excellent (>0.75), confirming the agreement between the experts was not due to chance. These indicated that all the items were considered relevant, therefore no items were removed during the content validation stage.

These were followed with face validation, which referred to the relevance evaluation of the content of the tool and its items. The degree to which respondents deemed the items on an evaluation tool to be appropriate is known as face validity. Therefore, the face validation respondents must be the people who were involved with the test, which in this case were the optometrists. Therefore, 10 optometrists from both the private and public sectors were approached and consented to participate in the face validation. The FVI for each item (I-FVI) ranges between 0.9 and 1.00, which was more than the expected value (>0.83). The average of the I-FVI scores for all the items on the scale (S-FVI/Ave) and the proportion of items on the scale that achieved comprehension and clarity was also more than 0.83 for all three domains. These indicated that the comprehensibility and clarity of sentence structure and language used in PECQ were satisfactory. This indicates the words and sentences of the constructed items in the PECQ were understood easily by optometrists.

The construct validity assessment showed that the PECQ had a good to excellent fit. The factor loading of each item in the PECQ was close to +1 to -1, indicating that the factor had strongly influenced the variable, whereas loadings close to 0 indicated that the factor had a weak influence on the variable. The total variance explained for each domain in this study was 78.69%, 68.77%, and 70.56%, respectively. This supported the conclusion that the individual items of the PECQ were important and relevant to measuring the contributing factors and barriers to optometrists' primary eye care practice.

The reliability of the PECQ as an instrument to evaluate the optometrist's scope of practice, challenges, and contributing factors to the practice of primary eye care in Malaysia was assessed using Cronbach’s alpha coefficient and test-retest reliability. The Cronbach’s alpha value of 0.971 was obtained, indicating an excellent agreement between the respondents. For test-retest reliability, all the optometrists completed the questionnaire for the second time, with few reminders in between. Therefore, the response rate achieved was 100%. The intraclass correlation coefficient was 0.948 (p < 0.01), indicating an excellent agreement. These indicate the PECQ is a reliable tool with excellent internal consistency.

Given these findings, the PECQ can be considered a valid and reliable instrument in assessing the optometrist's scope of practice, challenges, and contributing factors to the practice of primary eye care. Understanding the factors affecting the primary eye care practice measured across multiple domains may help develop targeted interventions and solutions that may increase the quality and delivery of primary eye care, thus reducing the waiting time at public hospitals and reducing the burden on ophthalmologists handling non-serious eye conditions.

The PECQ has potential applications in both the research setting and in clinical practice. Investigators can use this tool to survey optometrist practices and use this information to improve eye care services in Malaysia or a target country. The PECQ underwent a vigorous validation process, ensuring the PECQ's reliability and applicability.

Identifying the various contributing factors and challenges faced by optometrists to practice primary eye care using the PECQ will be one way to create a healthcare model for eye care and identify the solutions.

Study limitation

While this study provides valuable insights into developing and validating the PECQ, the study discovered a few limitations that future research should consider addressing. Although the study fulfilled the sample size for content and face validation, the sample used may not fully capture the diversity of samples. Future studies should consider diversified geographical locations, including varying cultural and socioeconomic status. Additionally, future studies should incorporate a larger sample for validation for improved generalizability of the findings.

## Conclusions

The newly developed PECQ is valid and reliable for assessing the contributing factors and challenges to practicing primary eye care by optometrists in Malaysia. The validity and reliability of this questionnaire were found to be satisfactory. The questionnaire will be able to gather baseline data on optometry practice in Malaysia. The information derived by using this questionnaire will be beneficial to the Ministry of Health in developing a healthcare model for eye care, thus reducing overcrowding in public hospitals.

## Appendices

The Primary Eye Care Questionnaire (PECQ)

**Table 12:** The Primary Eye Care Questionnaire (PECQ) items.

Scope of practice	
	In your opinion, which of the following is the optometrist’s scope for primary eye care?
Item 1	Refraction
Item 2	Dispensing
Item 3	Detection of ocular abnormalities
Item 4	Diagnosis of ocular abnormalities
Item 5	Diagnosis of visual abnormalities
Item 6	Management of ocular abnormalities
Item 7	Rehabilitation of the visual system
Item 8	As an optometrist, I do have a scope to practice primary eye care at my practice.
Readiness	
Item 9	In general, all optometrists are confident to practice primary eye care upon graduating with an optometry bachelor’s degree.
Item 10	As an optometrist, I do have a scope to practice primary eye care at my practice.
Item 11	I was confident to practice primary eye care upon graduating with an optometry bachelor’s degree.
Item 12	Optometrists are adequately trained to diagnose anterior segment ocular diseases.
Item 13	Optometrists are adequately trained to diagnose posterior segment ocular diseases.
Basic procedures	
	How often do you perform the following basic optometry procedures at your practice?
Item 14	History taking
Item 15	Visual acuity
Item 16	Pupillary reaction
Item 17	Stereopsis evaluation
Item 18	Color vision assessment
Item 19	Retinoscopy
Item 20	Subjective refraction
Item 21	Cycloplegic refraction
Item 22	Near refraction
Item 23	Keratometry evaluation
Item 24	Binocular vision assessment
Item 25	Slit lamp biomicroscopy assessment
Item 26	Fundus evaluation
Item 27	Tonometry
Item 28	Visual field assessment
Item 29	Contact lens assessment (e.g., soft/rigid gas permeable)
Item 30	Specialty contact lens assessment) (e.g., keratoconus/orthokeratology)
Item 31	Visual screening
Services	
	How often do you perform the following optometry procedures services at your practice?
Item 32	Pediatric eye assessment
Item 33	Binocular vision assessment
Item 34	Dry eye assessment
Item 35	Geriatric eye assessment
Item 36	Sports vision assessment
Item 37	Behavioral optometry assessment
Item 38	Low vision and rehabilitation assessment
Item 39	Electrodiagnostic assessment
Item 40	Preoperative assessment
Item 41	Postoperative assessment
Specialty procedures	
	How often do you use the following instruments for diagnostic evaluation purposes at your practice?
Item 42	Slit lamp biomicroscope
Item 43	Binocular indirect ophthalmoscope
Item 44	Fundus lens (Volk Lens, 90D)
Item 45	Fundus camera
Item 46	Goniolens
Item 47	Ocular coherence tomography (OCT)
Item 48	Biometry
Item 49	Corneal pachymetry
Item 50	Specular microscope
Item 51	Fluorescent staining
Item 52	Schirmer’s test
Item 53	Electrodiagnostic (visual evoked potential, electroretinogram)
Item 54	Visual field analyzer
Item 55	Goldmann Applanation Tonometer (GAT)
Item 56	How often do you use mydriatic diagnostic drugs in optometric examinations?
Item 57	How often do you use cycloplegic diagnostic drugs in optometric examinations?
Item 58	How often do you use anesthetic diagnostic drugs in optometric examinations?
Management	
	How frequently do you perform the following management at your practice?
Item 59	Spectacle dispensing
Item 60	Contact lens dispensing
Item 61	Prescribing vision therapy
Item 62	Prescribing low-vision aids
Item 63	Myopia management
Item 64	Color vision lens prescription
Item 65	Dry eye management
Item 66	Occupational vision therapy
Item 67	Referral to ophthalmologists
Item 68	Referral to non-ocular specialist
Referral	
	How often do you refer your patients for the following ocular conditions?
Item 68	Eye adnexa-related disease
Item 69	Eyelid-related symptoms
Item 70	Nasolacrimal abnormalities
Item 71	Strabismus
Item 72	Corneal conditions
Item 73	Crystalline lens condition besides cataract
Item 74	Cataract
Item 75	Retinal condition
Item 76	Retinopathy
Item 77	Vitreous condition
Item 78	Macular degeneration
Item 79	Glaucoma
Item 80	Visual impairment conditions
Item 81	Ocular infections
Item 82	Binocular vision
Item 83	Pediatric conditions
